# Dietary Habits among the JPHC Study Participants at Baseline Survey

**DOI:** 10.2188/jea.11.6sup_30

**Published:** 2007-11-30

**Authors:** Shoichiro Tsugane, Satoshi Sasaki, Minatsu Kobayashi, Yoshitaka Tsubono, Tomotaka Sobue

**Affiliations:** 1 Epidemiology and Biostatistics Division, National Cancer Center Research Institute East.; 2 Cancer Information and Epidemiology Division, National Cancer Center Research Institute.

**Keywords:** dietary habit, food frequency questionnaire, prospective study, baseline data

## Abstract

Dietary habit is closely associated with development of cancer and cardiovascular diseases, however little prospective evidence has been published for Japanese, whose dietary habit is substantially different from Western countries. Therefore, frequencies of food consumption, food preference, cooking method and acceptance of dietary advice were investigated at the baseline by two kinds of self-administered food frequency questionnaires. Dietary habits between urban and rural (Tokyo and Osaka vs. others), or between Okinawa and non-Okinawa revealed recognizable differences. The so-called westernized foods such as bread, beef and coffee were more consumed in the urban areas such as Tokyo and Osaka and also in Okinawa. The frequencies of salted food intake such as pickled vegetables and salted seafoods were remarkably low in Okinawa. Cooking methods for meats, seafoods and vegetables were also unique in Okinawa. No distinct geographical difference was shown in food preference and modification of dietary habit by dietary advice.

## INTRODUCTION

Animal experiments and observational evidences in human have provided many suggestions that dietary factors are of major importance in determining the risk of cancer. Doll and Peto^1^^)^ estimated the proportion of cancer deaths attributed to diet to be 35 % (range of acceptable estimates: 10 to 70) in 1981. Willett^2^^)^ also estimated recently that the proportion would be 32 % (20 to 42). The proportion varied by cancer site from 20 % in lung to 75 % in prostate. Such estimation was possible in the United States and other Western countries, because reasonable amount of epidemiological evidences in “diet and cancer” have been published in good quality. In particular, several prospective studies with large number of subjects have been conducted using validated dietary assessment methods^3^^)^.

On the contrary, little evidence has been available from prospective studies for Japanese whose dietary habits should be substantially different from Western countries. A Large-Scale Census-Based Cohort Study in Japan^4^^)^, commonly called as “Hirayama’s Cohort or Six Prefecture Cohort” which started in 1965, revealed one of initial evidences in the association between green-yellow vegetable intake and male lung cancer. Dietary habits, however, were assessed from only 8 items (rice, meat, fish and shellfish, milk and goat milk, green-yellow vegetables, pickles, soybean paste soup and green tea) with simple categories (Daily, Occasionally, Rarely, None, Uncertain).

In the Japan Public Health Center-based prospective Study on cancer and cardiovascular diseases (JPHC Study), we assessed the dietary habits of the subjects at the baseline survey using food frequency questionnaires. In this paper, we showed the method of dietary assessment and the results of frequencies of food consumption, food preference, cooking methods and acceptance of dietary advice by each study center with special reference to the geographical difference.

## METHODS

Dietary patterns were investigated by two kinds of self-administered baseline questionnaires in which frequency of food consumption, food preference, cooking methods and acceptance of dietary advice were included. The questionnaires used in JPHC Study Cohort I and II were slightly different with respect to food items, method of expression and frequency categories (Table 1).

**Table 1.** Food items, actual expression and categories in food frequency questionnaire used in JPHC study cohort I and II.

Food items	Actual expression in Cohort I	Categories	Actual expression in Cohort II	Categories	Portion size
Rice	Rice	(A)	Rice	(A)	
Miso soup	Miso soup	(B)	Miso soup	(B)	
Noodles	Noodles (exc. precooked noodles)	(C)	Noodles (exc. precooked noodles)	(E)	
Precooked noodles	Precooked noodles	(C)	Precooked noodles	(E)	
Bread	Bread (inc. pastry and cooked bread)	(C)	Bread	(E)	
Butter and margarine	Butter, margarine	(C)	Butter	(E)	Given
	–	–	Margarine	(E)	Given
Fruits	Fruits	(C)	Apple	(E)	Given
	–	–	Citrus fruits such as tangerine	(E)	Given
Green vegetables	Green vegetables such as spinach and garland chrysanthenam	(C)	Green vegetables (eg. spinach, Chinese chire)	(E)	Given
Yellow vegetables	Yellow vegetables such as carrot and pumpkin	(C)	Carrot	(E)	Given
Other vegetables	Other vegetables (eg. Chinese cabbage, radish, tomato, cucumber)	(C)	Tomato	(E)	Given
Dressing and mayonnaise	Dressing, mayonnaise	(C)	Dressing	(C)	
	–	–	Mayonnaise	(C)	
	–	–	Ketchup	(C)	
Pickled vegetables	Pickled vegetables	(C)	Nozawana-zuke,takana-zuke	(E)	
	–	–	Other pickled vegetables	(E)	
Mushrooms	Mushrooms	(C)	N/A	N/A	
Potatoes	Potatoes such as sweat potato and potato	(C)	Potato	(E)	Given
Seaweed	Seaweed such as laver, Wakame and Konbu	(C)	Seaweed (eg. Konbu, Wakame, Mozuku)	(E)	
Soybean and its products	Soybean and its products such as Tofu, Abura-age and Natto	(C)	Tofu	(E)	Given
	–	–	Fermented soybeans (“Natto”)	(E)	Given
Other beans	Other beans (eg. broad beans)	(C)	N/A	N/A	
Egg	Egg	(C)	Egg	(E)	Given
Milk and its products	Milk and its products	(C)	Yogurt	(E)	
Cheese	Cheese	(C)	Cheese	(E)	
Beef	Beef	(C)	Beef	(E)	Given
Pork	Pork	(C)	Pork	(E)	Given
Chicken	Chicken	(C)	Chicken	(E)	Given
Bacon,ham and sausage	Bacon,ham and sausage	(C)	Ham, sausage and bacon	(E)	
Liver	Liver	(C)	Liver and pluck (“Motsu”)	(E)	Given
Fresh fish	Fresh fish	(C)	Fresh fish (raw, cooked, grilled)	(E)	Given
Dried and salted fish	Dried fish (eg. Mezashi) and salted fish (eg. salted salmon)	(C)	Dried fish, salted salmon, pickeled fish in Sake lees	(E)	Given
Fish paste products	N/A	N/A	Fish paste products (Chikuwa, Kamaboko)	(E)	
Canned fish	N/A	N/A	Canned fish (Tuna, Sardin)	(E)	
Baby fish	N/A	N/A	Baby fish (“Shirasu”,“Jako”,“Mezashi”,“Tsukudani”)	(E)	Given
Salted roe	Salted roe (eg. Tarako, Sujiko and Ikura)	(C)	Salted roe, salted preserves (“Shio-kara”)	(E)	
Salted preserves and gonad roe	Salted preserves (Shio-kara) and salted gonad roe (Neri-uni)	(C)	–	–	

Japanese tea	Japanese tea (green tea)	(D)	Japanese tea (green tea)	(D)	
Chinese tea	Chinese tea (Oolong tea)	(D)	Chinese tea (Oolong tea)	(D)	
Black tea	Black tea	(D)	Black tea	(D)	
Other kind of tea	Other kind of tea	(D)	N/A	N/A	
Coffee	Coffee	(D)	Coffee	(D)	
Milk	Milk	(D)	Milk	(E)	Given
Carbonated beverages	Carbonated beverages (eg. cola and juice)	(D)	Cola and other carbonated beverages	(D)	
Pure fruit juices	Pure fruit juices	(D)	Pure fruit juices	(D)	
Vegetable juices	Vegetable juices	(D)	Vegetable juices	(D)	

Breakfast	Breakfast (exc. coffe or juice only)	(C)	N/A	N/A	
Evening meal at home	Evening meal at home	(C)	N/A	N/A	
Light meals	Light meals	(C)	N/A	N/A	
Foreign-style confectioneries	N/A	N/A	Foreign-style confectioneries	(E)	
Japanese-style confectioneries	N/A	N/A	Japanese-style confectioneries (“Manjyu”. “Yokan”)	(E)	
Fried and deep fried foods	Fried and deep fried foods	(C)	Fried and deep fried foods	(C)	

(A) No. of bowls/d; (B) 4 categories (Rarely, 1-2d/w, 3-4d/w, almost everyday) and No. of bowls/d for daily consumer; (C) 4 categories (Rarely, 1-2d/w, 3-4d/w, almost everyday); (D) 6 categoris (Rarely, 1-2d/w, 3-4d/w, 1-2 cups/d, 3-4 cups/d, 5- cups/d); (E) 5 categories (Never, Occasionally, 1-2d/w, 3-4d/w, almost everyday); N/A: not available

### Cohort I

The questionnaire used in Cohort I was a revised version of the questionnaire used in the cross-sectional epidemiological study for assessing cancer risks at the population level (ECOCANCER study), in which randomly selected men, aged 40s’, and their wives in the same five health center districts as Cohort I had been interviewed to know their lifestyles including dietary habits. The study design and the results of the food frequency questionnaire have been described elsewhere^5^^, ^^6^^, ^^7^^, ^^8^^)^. A total of 26 out of 38 food items in ECOCANCER study were consistent with JPHC Study Cohort I questionnaire and the other 12 food items, mostly sea foods and fruits, were asked in a different way in the Cohort I questionnaire.

For rice intake, the number of bowls (regular size) per day was asked for daily consumer. The intake frequency per week was asked using four categories: rarely (<1 day/week), 1-2 days/week, 3-4 days/week, almost everyday (5 days or more/week) for the following food items: miso (soybean paste) soup, noodles, precooked noodles, bread, butter and margarine, fruits, green vegetables, yellow vegetables, other vegetables, dressing and mayonnaise, pickled vegetables, mushrooms, potatoes, seaweed, soy beans and its products, other beans, egg, milk and its products, cheese, beef, pork, chicken, bacon, ham and sausages, liver, fresh fish, dried and salted fish, salted roe, salted preserves and gonad roe. Dietary habits, such as breakfast, an evening meal at home, light meals and fried and deep fried foods were also asked using the above 4 categories. For miso soup, typical quantity per day was further asked for those who responded to a category of almost everyday. The intake frequency of beverages was asked using six categories: rarely (less than 1 day/week), 1-2 days/week, 3-4 days/week, 1-2 cups/day, 3-4 cups/day, 5 cups or more/day, for the following food items: Japanese tea (green tea), Chinese tea (Oolong tea), black tea, other kind of tea, coffee, milk, carbonated beverages, pure fruit juice and vegetable juice.

The monthly frequency for each food item was calculated as four times the scores assigned to the four weekly frequency categories: rarely=0, 1-2 days/week=1.5, 3-4 days/week=3.5, almost everyday=6. For rice, miso soup and beverages, consumption was expressed in terms of daily frequency. When daily intake of these items was computed the number of bowls per day was used for rice and miso soup and the scores assigned to three daily frequency categories (1-2 cups/day=1.5, 3-4 cups/day=3.5, 5 cups or more/day=6) were used for subjects consuming beverages almost everyday. The scores of 0.5, 0.21, and 0 were assigned to subjects consuming beverages 3-4 days/week, 1-2 days/week, and rarely, respectively. The frequency of food consumption (percent) was also expressed in the indicated categories.

Food preferences were asked according to one of three categories: “like”, “intermediate” and “dislike” for the following preferences: deepfried/oily (“Kotteri”), hot (spicy) (“Karai”), salty (“Shio-kagen-no-koi”), sour (“Suppai”), sweet (“Amai”) and hot (high in temperature) (“Atsui”). The percent of “like” category responses was then calculated.

Most commonly used cooking methods for meats, seafood and vegetables were asked according to one of six categories: raw, boiled, grilled, deep fried, stir fried and others. The percent of responded subjects to each category was calculated.

Acceptance of dietary advice was also asked according to “no” or “yes” categories and the percent of “yes” and “no” were calculated.

Number of subjects used in this report is as following: 9,101 (4,229 men and 4,872 women) in Ninohe, 11,754 (5,471 men and 6,283 women) in Yokote, 10,887 (5,410 men and 5,477 women) in Saku, 10,453 (5,156 men and 5,297 women) in Ishikawa, and 7,004 (2,888 men and 4,116 women) in Katsushika. Number of missing values varied to each item of the questionnaire.

### Cohort II

The questionnaire used in JPHC Study Cohort II was a revised version of the questionnaire used in the JPHC Study Cohort I. In this questionnaire, specific food items were included with portion size such as apple (instead of fruits as a whole) and carrot (instead of yellow vegetables). Three choices for each portion size were given: half, same or 1.5 times more for a given portion size.

For rice, the number of bowls (regular size) per day was asked for daily consumer as same as in Cohort I. The intake frequency per week was asked according to one of four categories: rarely (<1 day/week), 1-2 days/week, 3-4 days/week, almost everyday (5 days or more/week) for the following food items: miso (soybean paste) soup, mayonnaise, dressing, ketchup and fried and deep fried dishes. For miso soup, typical quantity per day was further asked for those who responded to a category of almost everyday. The intake frequency per week was asked according to one of five categories for most food items: never, occasionally, 1-2 days/week, 3-4 days/week, almost everyday (5 days or more/week) for the following food items: noodles, precooked noodles, bread, butter, margarine, apple, citrus fruit, green vegetables, carrot, tomato, nozawana-zuke and takuan-zuke (pickled green-yellow vegetables), other pickled vegetables, potatoes, seaweed, tofu, fermented soybean, egg, milk, yogurt, cheese, beef, pork, chicken, ham, sausages and bacon, liver, fresh fish, dried and salted fish, fish paste products, canned fish, small fish, salted roe and salted preserves, foreign-style confectioneries and Japanese-style confectioneries. Questions about the intake frequency of beverages were the same as in Cohort I, except for deletion of “other kind of tea” and “milk”.

The monthly frequency for each food item was calculated as four times the scores assigned to the four or five weekly frequency categories: never, occasionally or rarely=0, 1-2 days/week=1.5, 3-4 days/week=3.5, almost everyday=6. For rice, miso soup and beverages, consumption was expressed in terms of daily frequency. Further calculations were the same as in Cohort I.

Food preferences were asked according to one of three categories: “like”, “general” and “dislike” for the following preferences: deepfried/oily (“Abura-koi”), salty (“Shio-kagen-no-koi”), sour (“Suppai”), sweet (“Amami-no-tsuyoi”) and hot (high in temperature) (“Atsui”). The percent of “like” category responses was then calculated.

Most commonly used cooking methods was asked according to one of five categories: raw, boiled, grilled, deep fried and stir fried for the following food groups: meats, seafood, green leafy vegetables and carrots. The percent of responses to each category was calculated.

Acceptance of dietary advice was also asked according to “no” or “yes” categories and the percent of accepted subjects was calculated. However, the question regarding the consumption of burnt part of fish or meat was slightly different, asking “Do you eat the burnt part of fish or meat?”, and “no” answer was used as an acceptance of dietary advice.

Number of subjects used in this report is as follows: 19,012 (9,453 men and 9,559 women) in Kasama, 3,165 (1,520 men and 1,645 women) in Kashiwazaki, 7,557 (3,561 men and 3,996 women) in Tosayamada, 11,199 (4,983 men and 6,216 women) in Arikawa, 10,048 (4,779 men and 5,269 women) in Miyako, 6,489 (2,907 men and 3,582 women) in Suita1, and 4,401 (2,055 men and 2,346 women) in Suita2. Number of missing values varied to each item of questionnaire.

## RESULTS

### Frequency of food consumption (Tables 2a, b, c, d)

﻿﻿Due to the different structure of the two questionnaires used in the Cohort I and II, geographical comparisons were separately done in each cohort, and their relative order was described. In general, the observed distribution was shifted toward the lower frequency categories when the category of “never” was added to four frequency categories as in Cohort II (“C” and “E” categories in Table 1). Consequently, the absolute values of mean frequency were systematically shifted to low in Cohort II for various food items.

**Table 2a.** Consumption of foods as a average number of days eaten per month* (left) and as a percent eaten in the category (right), cohort I, males.

Food or food group	Unit	Public Health Center District (Prefecture)					Category	Public Health Center District (Prefecture)				
	
		Ninohe (Iwate)	Yokote (Akita)	Saku (Nagano)	Ishikawa (Okinawa)	Katsushika (Tokyo)		Ninohe (Iwate)	Yokote (Akita)	Saku (Nagano)	Ishikawa (Okinawa)	Katsushika (Tokyo)
Rice	No. of bowls per day	4.2	4.0	3.9	2.6	3.0	≧ 3bowls/day	88.1	80.4	86.3	55.4	61.1
Miso soup	No. of days per month	22.8	22.5	21.1	18.1	18.1	≧ 5days/week	90.6	88.1	77.5	55.6	56.7
	No. of bowls per day	2.7	2.3	1.9	1.2	1.1						
Noodles	No. of days per month	7.0	6.9	7.6	7.8	8.7	≧ 1day/week	87.2	78.6	90.3	85.3	89.4
Precooked noodles	No. of days per month	2.7	2.1	2.8	3.2	2.9	≧ 1day/week	36.6	30.0	38.3	42.9	41.8
Bread	No. of days per month	5.2	4.6	6.0	8.7	9.2	≧ 1day/week	56.8	49.6	61.2	75.7	75.9
Butter and margarine	No. of days per month	4.2	3.3	5.0	7.0	7.4	≧ 1day/week	49.7	39.9	55.1	67.6	64.3
Fruits	No. of days per month	14.0	12.0	14.4	8.5	11.4	≧ 5days/week	33.9	21.6	34.8	9.7	22.0
Green vegetables	No. of days per month	13.5	15.4	13.3	14.7	11.3	≧ 5days/week	23.4	34.8	22.2	32.4	13.8
Yellow vegetables	No. of days per month	10.7	9.2	11.2	11.5	9.0	≧ 5days/week	14.3	9.4	16.2	17.2	7.0
Other vegetables	No. of days per month	14.9	17.0	15.9	12.2	14.1	≧ 5days/week	31.9	45.2	37.9	18.3	25.9
Dressing and mayonnaise	No. of days per month	6.0	7.2	7.6	6.1	8.2	≧ 1day/week	67.2	72.6	78.2	69.1	80.7
Pickled vegetables	No. of days per month	16.8	17.6	18.3	4.4	13.4	≧ 5days/week	52.7	56.9	60.8	4.2	32.3
Mushrooms	No. of days per month	6.8	6.8	7.5	4.2	6.2	≧ 1day/week	81.0	83.1	84.3	53.1	78.4
Potatoes	No. of days per month	8.2	7.3	11.9	7.8	7.2	≧ 5days/week	4.2	3.1	18.9	4.4	2.1
Seaweed	No. of days per month	13.2	13.6	12.8	10.6	11.6	≧ 5days/week	21.7	24.5	20.8	13.3	14.2
Soybean and its products	No. of days per month	17.8	17.7	13.3	12.6	12.5	≧ 5days/week	50.6	49.8	22.2	22.1	17.1
Other beans	No. of days per month	3.0	3.6	3.9	3.7	3.4	≧ 1day/week	35.5	44.1	48.9	43.6	47.5
Egg	No. of days per month	14.3	14.1	16.4	14.3	12.2	≧ 5days/week	30.1	28.3	43.4	30.7	18.9
Milk and its products	No. of days per month	13.4	12.8	14.1	10.6	11.6	≧ 5days/week	37.7	36.0	40.2	25.9	29.2
Cheese	No. of days per month	2.4	2.5	3.3	4.6	2.9	≧ 1day/week	31.0	32.3	42.0	54.0	38.1
Beef	No. of days per month	3.2	4.6	4.1	7.1	6.2	≧ 1day/week	42.9	61.6	54.0	86.3	82.0
Pork	No. of days per month	8.0	8.1	8.8	7.7	7.3	≧ 1day/week	90.1	89.9	92.4	88.9	88.0
Chicken	No. of days per month	7.7	7.0	7.0	6.9	5.9	≧ 1day/week	88.1	84.5	82.4	86.5	79.2
Bacon,ham and sausage	No. of days per month	5.4	6.4	6.2	7.4	5.7	≧ 1day/week	65.7	75.0	73.9	82.0	73.3
Liver	No. of days per month	4.2	3.5	2.6	3.4	2.7	≧ 1day/week	56.0	46.4	36.9	49.4	41.5
Fresh fish	No. of days per month	12.6	13.9	10.4	7.3	9.2	≧ 1day/week	95.8	97.2	93.8	83.2	92.4
Dried fish	No. of days per month	9.5	9.2	9.1	2.5	6.6	≧ 1day/week	91.2	91.5	92.3	33.3	82.4
Salted roe	No. of days per month	5.8	7.9	4.3	0.9	4.0	≧ 1day/week	69.9	84.5	57.7	11.9	55.8
Salted preserves	No. of days per month	4.3	4.9	3.8	1.0	2.4	≧ 1day/week	56.0	62.7	50.1	12.3	35.0

Japanese tea	No. of cups per day	1.9	2.8	3.4	2.2	1.9	≧ 5days/week	55.7	71.3	82.9	51.9	60.2
Chinese tea	No. of cups per day	0.2	0.3	0.3	0.6	0.7	≧ 1day/week	22.9	28.4	27.4	45.2	58.8
Black tea	No. of cups per day	0.1	0.1	0.1	0.2	0.1	≧ 1day/week	11.0	12.6	16.7	24.5	24.8
Other kind of tea	No. of cups per day	0.4	0.6	0.4	1.0	0.4	≧ 1day/week	28.1	30.4	23.9	41.8	24.1
Coffee	No. of cups per day	1.0	0.7	0.8	1.2	1.3	≧ 5days/week	36.8	25.9	30.1	46.1	50.2
Milk	No. of cups per day	0.9	0.7	1.0	0.7	0.7	≧ 5days/week	39.3	37.6	41.7	28.0	29.5
Carbonated beverages	No. of cups per day	0.3	0.5	0.2	0.4	0.3	≧ 1day/week	48.4	61.3	32.7	57.6	48.7
Pure fruit juices	No. of cups per day	0.2	0.2	0.1	0.2	0.2	≧ 1day/week	44.4	49.4	34.2	40.1	43.3
Vegetable juices	No. of cups per day	0.2	0.2	0.1	0.2	0.2	≧ 1day/week	26.0	26.0	20.2	32.5	26.3

Breakfast	No. of days per month	21.7	22.0	22.0	17.4	19.5	≧ 5days/week	87.9	89.5	89.7	65.7	75.6
Evening meal at home	No. of days per month	22.8	22.6	22.6	22.3	20.9	≧ 5days/week	91.5	89.4	89.4	86.9	78.1
Light meals	No. of days per month	8.6	7.8	8.1	8.4	6.9	≧ 5days/week	22.8	20.6	20.8	19.7	14.1
Fried and deep fried foods	No. of days per month	12.0	11.5	13.0	17.3	11.9	≧ 5days/week	15.0	10.8	18.2	49.0	12.5

* Mean of ordered categories

**Table 2b.** Consumption of foods as a average number of days eaten per month* (left) and as a percent eaten in the category (right), cohort I, females.

Food or food group	Unit	Public Health Center District (Prefecture)					Category	Public Health Center District (Prefecture)				
	
		Ninohe (Iwate)	Yokote (Akita)	Saku (Nagano)	Ishikawa (Okinawa)	Katsushika (Tokyo)		Ninohe (Iwate)	Yokote (Akita)	Saku (Nagano)	Ishikawa (Okinawa)	Katsushika (Tokyo)
Rice	No. of bowls per day	3.3	3.0	2.9	2.2	2.4	≧ 3bowls/day	81.5	72.3	74.6	37.6	42.6
Miso soup	No. of days per month	22.4	21.9	20.1	17.8	18.1	≧ 5days/week	87.8	84.4	72.0	54.5	56.1
Miso soup	No. of bowls per day	2.3	2.0	1.5	1.2	1.0						
Noodles	No. of days per month	6.9	6.4	7.2	6.8	7.0	≧ 1day/week	87.0	76.5	90.2	81.0	85.4
Precooked noodles	No. of days per month	1.8	1.3	1.9	2.3	2.0	≧ 1day/week	24.6	17.8	26.9	31.6	30.3
Bread	No. of days per month	7.4	6.9	8.5	11.3	12.0	≧ 1day/week	72.7	68.7	79.7	88.0	89.3
Butter and margarine	No. of days per month	5.4	4.2	6.6	8.3	10.0	≧ 1day/week	58.2	46.0	67.7	74.0	81.4
Fruits	No. of days per month	18.7	15.9	19.5	10.8	16.9	≧ 5days/week	60.3	39.3	64.6	16.6	47.7
Green vegetables	No. of days per month	14.9	17.5	14.3	16.0	13.7	≧ 5days/week	29.7	47.9	25.9	39.8	22.3
Yellow vegetables	No. of days per month	13.4	11.8	14.7	14.0	12.8	≧ 5days/week	23.2	15.7	31.1	27.1	16.9
Other vegetables	No. of days per month	16.6	19.1	18.0	13.8	17.9	≧ 5days/week	41.4	59.0	50.7	24.9	48.0
Dressing and mayonnaise	No. of days per month	8.0	9.6	10.1	6.8	10.4	≧ 1day/week	81.8	86.0	91.6	74.7	91.3
Pickled vegetables	No. of days per month	18.0	19.3	20.2	5.0	15.3	≧ 5days/week	61.0	67.6	73.3	5.7	43.5
Mushrooms	No. of days per month	7.5	7.9	8.5	5.4	8.9	≧ 1day/week	83.2	86.5	87.6	62.7	92.1
Potatoes	No. of days per month	10.2	9.4	14.4	9.8	10.5	≧ 5days/week	7.7	6.3	28.1	8.5	7.3
Seaweed	No. of days per month	14.3	14.9	14.7	12.6	14.2	≧ 5days/week	26.6	30.7	29.8	20.3	24.8
Soybean and its products	No. of days per month	19.1	19.3	14.8	14.3	14.9	≧ 5days/week	60.3	62.7	28.8	29.4	27.6
Other beans	No. of days per month	3.3	4.1	4.7	4.3	4.2	≧ 1day/week	38.4	48.8	56.5	47.9	55.9
Egg	No. of days per month	14.4	14.7	16.7	14.5	13.9	≧ 5days/week	29.0	31.5	44.7	32.8	24.8
Milk and its products	No. of days per month	15.3	13.9	16.0	13.1	16.3	≧ 5days/week	47.3	41.2	48.4	36.8	50.5
Cheese	No. of days per month	2.3	1.9	3.4	4.9	3.7	≧ 1day/week	30.1	26.0	42.9	56.8	47.5
Beef	No. of days per month	2.6	3.7	3.1	6.8	6.1	≧ 1day/week	35.1	50.0	40.7	83.1	79.7
Pork	No. of days per month	8.0	7.8	9.4	7.5	8.7	≧ 1day/week	87.3	84.1	90.7	87.2	91.3
Chicken	No. of days per month	8.3	8.0	8.1	7.2	7.1	≧ 1day/week	90.6	88.7	87.4	86.9	87.3
Bacon,ham and sausage	No. of days per month	5.5	6.4	6.7	7.2	6.7	≧ 1day/week	65.3	71.2	76.1	80.6	78.9
Liver	No. of days per month	3.7	2.5	2.1	3.7	2.3	≧ 1day/week	49.2	33.1	29.9	52.4	34.2
Fresh fish	No. of days per month	13.2	15.5	11.2	7.2	10.0	≧ 1day/week	95.5	97.5	93.2	81.2	94.2
Dried fish	No. of days per month	9.8	10.3	9.9	2.6	7.5	≧ 1day/week	91.1	93.6	94.2	34.5	87.3
Salted roe	No. of days per month	5.4	8.0	4.3	0.8	4.5	≧ 1day/week	65.4	82.9	57.0	10.4	59.5
Salted preserves	No. of days per month	3.0	3.7	2.4	0.6	1.6	≧ 1day/week	40.9	48.1	33.8	8.1	23.2

Japanese tea	No. of cups per day	1.6	2.9	3.4	2.2	2.3	≧ 5days/week	48.7	71.6	81.1	54.7	66.0
Chinese tea	No. of cups per day	0.4	0.4	0.4	0.7	0.7	≧ 1day/week	26.2	31.4	29.5	44.1	53.1
Black tea	No. of cups per day	0.1	0.1	0.1	0.2	0.2	≧ 1day/week	14.6	16.1	19.9	25.1	34.9
Other kind of tea	No. of cups per day	0.5	0.7	0.7	1.0	0.6	≧ 1day/week	33.1	36.1	32.5	43.2	34.7
Coffee	No. of cups per day	0.9	0.6	0.7	1.2	1.2	≧ 5days/week	37.9	24.9	29.2	51.8	50.0
Milk	No. of cups per day	1.0	0.9	1.0	0.8	1.0	≧ 5days/week	45.2	39.4	46.9	36.9	47.2
Carbonated beverages	No. of cups per day	0.2	0.2	0.1	0.3	0.2	≧ 1day/week	32.2	43.7	19.6	44.9	32.5
Pure fruit juices	No. of cups per day	0.2	0.2	0.1	0.2	0.2	≧ 1day/week	44.9	48.2	31.8	40.9	42.8
Vegetable juices	No. of cups per day	0.2	0.2	0.1	0.2	0.2	≧ 1day/week	16.7	17.2	12.4	27.5	18.8

Breakfast	No. of days per month	21.8	21.7	21.9	17.8	21.4	≧ 5days/week	88.3	88.1	89.2	67.1	84.9
Evening meal at home	No. of days per month	23.5	23.5	23.5	23.1	23.3	≧ 5days/week	97.1	96.8	96.6	93.9	94.9
Light meals	No. of days per month	13.0	12.9	13.0	11.2	11.2	≧ 5days/week	38.6	39.4	38.8	29.9	26.8
Fried and deep fried foods	No. of days per month	12.5	11.9	14.1	18.2	12.7	≧ 5days/week	16.4	12.1	21.9	56.6	14.4

* Mean of ordered categories

**Table 2c.** Consumption of foods as a average number of days eaten per month* (left) and as a percent eaten in the category (right), cohort II, males.

Food or food group	Unit	Public Health Center District (Prefecture)							Category	Public Health Center District (Prefecture)						
	
		Kasama (Ibaragi)	Kashiwazaki (Niigata)	Tosayamada (Kochi)	Arikawa (Nagasaki)	Miyako (Okinawa)	Suita1 (Osaka)	Suita2 (Osaka)		Kasama (Ibaragi)	Kashiwazaki (Niigata)	Tosayamada (Kochi)	Arikawa (Nagasaki)	Miyako (Okinawa)	Suita1 (Osaka)	Suita2 (Osaka)
Rice (a)	No. of bowls per day	3.3	4.0	3.9	3.2	2.9	2.8	2.8	≧ 3bowls/day	76.6	89.9	81.7	75.8	72.3	55.6	55.2
Miso soup	No. of days per month	21.0	22.2	17.1	20.7	19.9	14.8	13.8	≧ 5days/week	77.8	86.4	51.8	74.1	69.6	37.6	32.5
Miso soup (a)	No. of bowls per day	1.7	2.1	0.8	1.4	1.7	0.7	0.7								
Noodles	No. of days per month	4.6	3.4	3.3	5.1	4.0	6.4	6.2	≧ 1day/week	50.6	39.4	40.8	54.5	43.0	65.8	62.7
Precooked noodles	No. of days per month	1.5	1.3	1.5	1.7	2.3	1.8	1.2	≧ 1day/week	18.8	15.6	18.2	19.1	25.1	23.1	15.8
Bread	No. of days per month	4.4	3.6	6.7	5.9	5.7	11.5	12.5	≧ 1day/week	37.9	30.5	47.0	40.7	44.1	70.1	69.8
Butter	No. of days per month	1.1	1.2	1.1	1.4	1.6	2.8	2.4	≧ 1day/week	12.0	12.8	10.3	12.4	15.1	24.1	19.0
Margarine	No. of days per month	1.9	2.1	2.5	2.5	1.8	6.5	6.2	≧ 1day/week	16.9	17.4	18.1	17.7	15.0	43.0	38.7
Apple	No. of days per month	4.9	3.8	5.0	4.2	2.9	3.2	4.6	≧ 5days/week	8.0	5.8	8.8	7.5	3.5	4.3	8.9
Citrus fruits	No. of days per month	8.7	5.9	11.3	8.1	5.1	4.3	5.9	≧ 5days/week	19.1	10.5	29.5	18.4	8.4	5.2	10.1
Green vegetables	No. of days per month	10.1	12.3	11.9	8.6	12.5	8.5	9.7	≧ 5days/week	17.4	28.4	22.5	13.8	30.8	10.9	14.6
Carrot	No. of days per month	6.8	7.7	5.6	7.3	9.4	6.1	6.3	≧ 5days/week	8.6	12.3	5.5	10.7	17.1	6.1	7.2
Tomato	No. of days per month	3.2	3.4	4.5	3.5	2.6	5.2	6.2	≧ 5days/week	2.5	2.8	4.3	3.9	3.0	4.6	8.3
Dressing	No. of days per month	4.3	3.7	4.0	3.5	4.4	4.6	4.4	≧ 1day/week	54.4	43.6	45.1	40.7	52.0	54.6	51.6
Mayonnaise	No. of days per month	5.5	5.8	5.5	7.1	4.9	6.1	5.2	≧ 1day/week	68.9	68.6	63.2	75.3	59.9	69.9	60.4
Ketchup	No. of days per month	2.3	1.8	2.1	3.2	3.9	2.5	2.4	≧ 1day/week	32.6	24.3	29.3	41.7	49.1	36.3	33.9
Nozawana-zuke and takana-zuke	No. of days per month	2.7	7.7	1.9	4.2	1.2	1.8	2.4	≧ 5days/week	4.7	17.3	3.1	8.8	2.0	1.8	2.9
Other pickled vegetables	No. of days per month	11.7	11.9	9.5	9.5	3.2	7.8	8.5	≧ 5days/week	34.6	33.0	24.7	26.3	7.1	17.6	20.2
Potato	No. of days per month	5.1	6.8	4.2	6.4	3.7	5.1	5.4	≧ 5days/week	3.0	9.2	1.4	4.4	3.1	1.7	2.4
Seaweed	No. of days per month	6.8	8.0	8.1	7.7	7.1	6.0	7.1	≧ 1day/week	62.4	65.5	65.9	62.2	59.4	57.3	63.6
Tofu	No. of days per month	8.9	11.7	13.2	11.6	13.4	8.9	10.0	≧ 5days/week	11.7	22.8	30.4	24.9	32.5	10.5	14.5
Fermented soybeans	No. of days per month	9.6	6.8	1.6	1.7	1.2	2.6	2.8	≧ 5days/week	17.5	9.7	2.1	2.5	1.5	2.3	3.4
Egg	No. of days per month	12.1	14.0	12.9	12.1	12.9	13.0	11.1	≧ 5days/week	26.7	35.2	27.7	27.9	31.6	29.4	22.4
Yogurt	No. of days per month	2.2	1.2	1.2	1.1	1.2	1.7	2.4	≧ 1day/week	17.6	10.1	9.0	8.2	11.3	13.9	17.3
Cheese	No. of days per month	1.8	1.8	1.1	0.8	1.0	1.8	2.4	≧ 1day/week	17.6	17.1	10.1	7.9	10.7	19.3	21.9
Beef	No. of days per month	1.7	1.0	3.7	2.4	2.0	4.9	4.4	≧ 1day/week	23.5	12.5	52.1	31.0	24.7	66.8	61.7
Pork	No. of days per month	4.9	6.2	3.4	3.9	5.4	4.1	3.3	≧ 1day/week	56.6	63.8	45.8	44.2	53.4	57.7	47.3
Chicken	No. of days per month	3.2	2.7	4.2	3.4	2.9	3.7	3.5	≧ 1day/week	41.9	33.4	55.1	41.8	36.3	52.7	49.6
Ham. sausage and bacon	No. of days per month	2.6	2.8	1.4	2.0	2.9	3.6	2.9	≧ 1day/week	30.1	30.9	17.4	21.7	29.6	40.5	30.0
Liver	No. of days per month	0.8	0.8	0.5	0.5	0.8	0.4	0.3	≧ 1day/week	11.0	9.6	7.2	6.2	9.9	5.6	4.8
Fresh fish	No. of days per month	9.0	8.5	13.5	13.6	10.3	7.9	9.2	≧ 1day/week	79.6	72.0	90.3	84.9	75.3	81.9	87.0
Dried fish	No. of days per month	4.6	4.1	6.1	4.3	0.7	2.5	3.0	≧ 1day/week	50.0	42.8	58.0	39.8	7.2	31.3	35.6
Fish paste products	No. of days per month	1.8	3.5	4.2	3.9	2.1	2.8	3.0	≧ 1day/week	23.2	36.9	46.6	39.0	22.7	35.7	37.6
Canned fish	No. of days per month	1.2	1.4	0.7	0.7	4.9	0.7	0.5	≧ 1day/week	15.8	17.2	8.9	8.7	42.0	10.7	6.8
Baby fish	No. of days per month	5.1	2.2	8.6	2.2	2.1	3.9	5.3	≧ 1day/week	45.4	25.1	68.7	19.9	18.5	54.9	50.3
Salted roe and salted preserves	No. of days per month	1.9	2.7	0.5	1.0	0.5	1.0	1.0	≧ 1day/week	21.0	28.1	5.4	9.0	5.2	12.9	12.8

Japanese tea	No. of cups per day	3.4	3.0	2.7	3.2	2.2	2.0	2.5	≧ 5days/week	87.1	80.7	82.6	86.9	60.7	68.9	78.6
Chinese tea	No. of cups per day	0.3	0.2	0.2	0.3	0.8	0.6	0.6	≧ 1day/week	36.3	24.5	25.0	28.0	65.4	55.7	45.6
Black tea	No. of cups per day	0.1	0.04	0.1	0.1	0.1	0.2	0.2	≧ 1day/week	20.3	10.6	12.3	14.2	24.1	31.4	30.0
Coffee	No. of cups per day	1.0	0.9	1.4	1.1	1.1	2.0	1.5	≧ 5days/week	33.9	33.6	53.8	37.5	43.3	74.6	60.5
Carbonated beverages	No. of cups per day	0.2	0.2	0.1	0.2	0.3	0.2	0.1	≧ 1day/week	33.5	33.7	25.6	34.7	52.1	36.4	26.7
Milk	No. of days per month	10.1	12.4	9.9	8.2	10.1	10.1	11.7	≧ 5days/week	31.2	42.3	32.4	24.9	30.6	30.9	38.4
Pure fruit juices	No. of cups per day	0.2	0.1	0.1	0.2	0.2	0.2	0.2	≧ 1day/week	43.1	34.9	33.1	43.6	50.3	44.9	43.2
Vegetable juices	No. of cups per day	0.1	0.1	0.1	0.1	0.2	0.1	0.2	≧ 1day/week	30.4	17.7	18.0	22.8	42.4	24.5	26.4

Fried and deep fried foods	No. of days per month	10.0	12.1	9.3	11.4	16.1	10.1	8.9	≧ 5days/week	8.4	17.3	6.7	12.4	43.3	7.9	5.3
Foreign-style confectioneries	No. of days per month	1.1	0.9	1.2	1.5	1.6	1.4	1.1	≧ 1day/week	13.2	10.4	13.5	14.9	14.9	16.2	14.1
Japanese-style confectioneries	No. of days per month	2.3	2.1	1.9	2.4	1.5	1.2	2.2	≧ 1day/week	24.6	20.6	20.9	23.5	13.7	15.2	24.2

* Mean of ordered categories

**Table 2d.** Consumption of foods as a average number of days eaten per month* (left) and as a percent eaten in the category (right), cohort II, females.

Food or food group	Unit	Public Health Center District (Prefecture)							Category	Public Health Center District (Prefecture)						
	
		Kasama (Ibaragi)	Kashiwazaki (Niigata)	Tosayamada (Kochi)	Arikawa (Nagasaki)	Miyako (Okinawa)	Suita1 (Osaka)	Suita2 (Osaka)		Kasama (Ibaragi)	Kashiwazaki (Niigata)	Tosayamada (Kochi)	Arikawa (Nagasaki)	Miyako (Okinawa)	Suita1 (Osaka)	Suita2 (Osaka)
Rice	No. of bowls per day	2.7	3.0	3.0	2.7	2.5	2.3	2.3	≧ 3bowls/day	64.8	82.7	67.5	65.8	55.9	37.1	37.5
Miso soup	No. of days per month	20.6	21.6	16.6	19.8	19.2	14.0	12.6	≧ 5days/week	74.3	83.3	48.3	67.2	65.3	30.9	25.8
Miso soup	No. of bowls per day	1.5	1.6	0.7	1.2	1.4	0.6	0.6								
Noodles	No. of days per month	3.7	3.0	3.1	4.9	3.2	4.4	4.5	≧ 1day/week	44.3	35.4	40.0	53.5	36.3	56.1	51.3
Precooked noodles	No. of days per month	0.9	0.6	0.7	0.7	1.2	0.7	0.5	≧ 1day/week	11.1	7.1	9.5	8.0	13.6	10.2	6.3
Bread	No. of days per month	6.6	5.8	10.1	8.1	8.1	16.0	15.7	≧ 1day/week	52.9	44.3	65.8	52.6	56.0	86.8	81.5
Butter	No. of days per month	1.4	1.2	1.4	1.8	2.2	3.2	2.4	≧ 1day/week	15.2	12.9	12.1	14.8	18.8	26.9	18.8
Margarine	No. of days per month	3.0	3.1	4.4	3.7	2.7	9.9	8.9	≧ 1day/week	25.4	23.9	30.1	25.4	20.9	60.4	51.6
Apple	No. of days per month	8.7	6.6	8.7	6.7	4.6	6.1	7.5	≧ 5days/week	17.1	12.3	17.7	13.1	7.0	10.8	16.5
Citrus fruits	No. of days per month	14.5	11.1	16.6	12.8	8.0	9.1	10.7	≧ 5days/week	42.1	26.2	53.2	35.2	16.2	17.4	24.9
Green vegetables	No. of days per month	12.7	14.1	14.3	9.9	14.2	12.3	13.3	≧ 5days/week	25.6	34.7	30.8	16.4	38.6	21.1	26.0
Carrot	No. of days per month	10.9	12.9	8.7	10.5	12.2	10.7	10.5	≧ 5days/week	18.8	28.1	10.2	18.4	26.2	13.8	15.7
Tomato	No. of days per month	4.3	4.3	6.0	3.8	3.3	7.6	8.5	≧ 5days/week	3.9	4.1	7.2	3.9	4.6	9.3	12.7
Dressing	No. of days per month	6.0	5.5	5.5	4.4	5.1	5.8	5.2	≧ 1day/week	69.9	62.0	58.4	48.5	58.4	66.3	59.1
Mayonnaise	No. of days per month	6.7	7.5	6.3	7.1	5.0	6.4	5.2	≧ 1day/week	80.9	86.1	72.1	79.0	61.2	76.0	64.5
Ketchup	No. of days per month	3.1	2.7	2.7	3.7	4.4	3.4	2.8	≧ 1day/week	44.3	34.9	38.5	49.0	54.1	50.3	42.0
Nozawana-zuke and takana-zuke	No. of days per month	2.4	8.1	1.7	4.4	1.2	1.5	2.0	≧ 5days/week	4.7	20.2	2.9	10.5	2.6	1.6	2.8
Other pickled vegetables	No. of days per month	14.2	13.3	10.3	10.5	3.9	8.5	8.9	≧ 5days/week	45.8	42.1	27.9	32.0	9.3	20.3	23.7
Potato	No. of days per month	7.2	9.5	6.0	8.0	4.9	8.4	8.0	≧ 5days/week	5.3	13.6	2.3	6.3	4.6	4.5	2.3
Seaweed	No. of days per month	8.8	10.1	10.7	9.3	8.6	8.8	9.6	≧ 1day/week	74.5	77.3	79.8	70.5	67.1	76.7	79.1
Tofu	No. of days per month	10.6	12.6	14.7	12.6	15.1	10.7	11.5	≧ 5days/week	15.3	24.4	36.3	27.0	40.8	13.0	17.2
Fermented soybeans	No. of days per month	11.0	7.0	1.8	2.0	1.6	3.6	3.5	≧ 5days/week	22.1	10.6	2.3	2.7	2.8	3.4	4.2
Egg	No. of days per month	12.8	14.4	13.6	12.1	12.1	14.6	10.8	≧ 5days/week	28.7	36.9	30.6	26.9	29.2	34.3	20.1
Yogurt	No. of days per month	4.5	2.3	2.2	1.6	1.8	4.1	5.0	≧ 1day/week	34.6	18.7	16.6	12.8	16.0	34.3	34.7
Cheese	No. of days per month	2.3	1.5	1.4	0.8	1.4	3.2	3.4	≧ 1day/week	21.6	15.3	12.1	7.9	13.2	30.5	28.1
Beef	No. of days per month	1.6	0.6	3.8	2.1	1.8	5.4	4.4	≧ 1day/week	23.2	8.0	53.3	26.9	22.2	73.0	61.3
Pork	No. of days per month	5.6	7.3	3.9	4.3	5.2	5.1	3.9	≧ 1day/week	62.7	71.7	51.7	47.3	52.2	70.1	54.5
Chicken	No. of days per month	3.8	2.7	4.7	3.8	2.7	4.3	3.9	≧ 1day/week	49.6	34.6	60.5	44.9	34.9	63.2	55.0
Ham, sausage and bacon	No. of days per month	2.8	3.0	1.5	1.7	2.8	4.0	2.4	≧ 1day/week	32.4	32.9	17.9	19.6	28.0	43.5	26.8
Liver	No. of days per month	0.6	0.6	0.5	0.5	0.8	0.3	0.3	≧ 1day/week	7.4	6.5	6.6	6.4	10.9	3.8	4.0
Fresh fish	No. of days per month	9.4	9.6	13.7	11.1	9.3	8.4	9.6	≧ 1day/week	81.3	78.3	92.1	77.7	72.8	86.3	87.0
Dried fish	No. of days per month	5.4	4.7	6.0	3.8	0.8	3.0	3.3	≧ 1day/week	57.7	49.3	57.4	36.3	7.6	39.5	39.9
Fish paste products	No. of days per month	2.4	5.2	4.7	4.2	2.3	3.1	3.0	≧ 1day/week	30.1	49.6	50.9	41.3	24.7	40.4	37.2
Canned fish	No. of days per month	1.7	2.1	0.8	0.8	6.7	0.9	0.6	≧ 1day/week	21.1	25.2	11.5	9.9	93.6	13.7	8.8
Baby fish	No. of days per month	7.0	3.2	10.4	2.8	2.8	6.0	7.3	≧ 1day/week	56.5	34.0	76.0	23.7	23.3	56.8	62.7
Salted roe and salted preserves	No. of days per month	1.4	2.0	0.3	0.5	0.4	0.8	0.7	≧ 1day/week	16.5	21.1	3.4	5.0	4.1	10.0	8.7

Japanese tea	No. of cups per day	3.7	3.2	3.1	3.5	2.5	2.4	3.0	≧ 5days/week	89.0	81.7	88.0	89.0	67.6	73.9	80.8
Chinese tea	No. of cups per day	0.4	0.2	0.2	0.4	0.9	0.7	0.7	≧ 1day/week	37.0	20.0	22.0	26.2	62.7	48.1	43.4
Black tea	No. of cups per day	0.1	0.1	0.1	0.1	0.1	0.3	0.3	≧ 1day/week	26.8	12.4	21.9	19.6	22.7	48.1	45.2
Coffee	No. of cups per day	0.8	0.7	1.3	0.8	1.1	1.6	1.2	≧ 5days/week	29.2	31.3	57.3	30.3	45.8	67.8	55.0
Carbonated beverages	No. of cups per day	0.1	0.1	0.1	0.1	0.2	0.1	0.1	≧ 1day/week	19.5	15.4	10.9	17.9	33.8	18.4	13.7
Milk	No. of days per month	13.1	14.5	11.8	9.8	12.8	15	15.6	≧ 5days/week	43.0	51.5	39.4	30.5	41.8	50.2	54.2
Pure fruit juices	No. of cups per day	0.2	0.1	0.1	0.2	0.2	0.2	0.2	≧ 1day/week	44.2	32.4	32.2	42.3	51.2	46.6	41.4
Vegetable juices	No. of cups per day	0.1	0.1	0.1	0.1	0.2	0.1	0.2	≧ 1day/week	23.2	10.0	15.3	17.3	38.5	20.1	24.2

Fried and deep fried foods	No. of days per month	10.2	14.1	9.9	11.6	16.9	9.8	8.5	≧ 5days/week	8.2	26.9	7.5	13.2	48.2	6.4	3.8
Foreign-style confectioneries	No. of days per month	1.7	1.3	1.9	1.8	2.3	2.6	1.7	≧ 1day/week	19.0	13.7	21.6	17.6	19.1	32.1	20.0
Japanese-style confectioneries	No. of days per month	4.1	3.3	3.3	3.0	1.8	3.3	4.3	≧ 1day/week	40.4	32.6	33.9	29.3	16.3	38.1	42.4

* Mean of ordered categories

The mean frequency of rice consumption was relatively lower in Tokyo, two areas in Osaka, and Okinawa (Ishikawa) than in the other areas. Consumption of bread showed converse geographical difference. Miso soup showed the same regional difference as rice. Butter and margarine showed, on the other hand, the same pattern as bread. The mean frequency for fruit intake was relatively low in two areas in Okinawa, and slightly lower in Tokyo and Osaka than in the other areas. The mean frequency for green and yellow vegetables was low in Tokyo, Osaka, and Nagasaki. The pickled vegetables were frequently taken in the areas of northern Japan such as Iwate, Akita, Nagano, and Niigata. It was considerably low in Okinawa. For soybean and its products, the result was different between Cohort I and II. Two areas in Tohoku such as Iwate and Akita in Cohort I showed a higher mean frequency than the other areas. In Cohort II in which “tofu” was categorized as an independent food, Kochi and Okinawa, which are locate in the southern part of Japan, showed a higher mean frequency than the other areas. The mean for fermented soybeans, included only in Cohort II as an independent food, was considerably high in Ibaragi. Egg intake did not show any noticeable regional difference. The mean of cheese intake was high in Tokyo and Okinawa (Ishikawa). Among meats, only beef consumption showed a relative regional difference. It was high in Tokyo, Osaka, and Okinawa (Ishikawa). As for the sea foods, mean intake frequency of dried and salted fish was low in Osaka and two areas in Okinawa, but those of fresh fish was high in Iwate, Akita, Kochi, and Nagasaki. In Okinawa, mean frequency of other salted seafoods, such as salted roes, salted preserves, and salted gonad paste was also lower than the other areas. The mean intake frequency of canned fish, which was only asked in the Cohort II questionnaire, was high in Miyako, Okinawa. Among beverages, the mean frequency of Japanese tea was relatively high in Nagano and Ibaragi. On the other hand, the mean frequency of coffee was high in Tokyo, Osaka, and Okinawa (Ishikawa). Milk did not show any regional difference.

No consistent difference in mean consumption patterns was observed between men and women. For certain foods, such as mean frequency of fruit and green yellow vegetables, consumption was generally higher in women than in men.

The percent frequency of food consumption showed similar regional patterns as the mean frequencies of monthly food consumption.

### Food preference (Tables 3a, b, c, d)

﻿﻿“Deep fried/Oily” or “Oily” was preferred in Okinawa compared to the other areas. No remarkable regional difference was observed for the other food preference. Sex difference was not observed.

**Table 3a.** Food Preferences (percent), cohort I, males.

	Public Health Center District (Prefecture)				

	Ninohe (Iwate)	Yokote (Akita)	Saku (Nagano)	Ishikawa (Okinawa)	Katsushika (Tokyo)
Deepfried/Oily (“Kotteri”)	23.3	20.9	26.9	33.7	24.7
Hot	29.6	27.9	32.8	19.8	36.9
Salty	22.4	21.6	23.3	13.8	20.8
Sour	25.3	18.1	21.3	14.4	22.4
Sweet (eg. confectionery)	27.1	22.9	22.9	30.3	24.4
Hot (temperature)	30.6	22.1	22.5	28.7	24.6

**Table 3b.** Food Preferences (percent), cohort I, females.

	Public Health Center District (Prefecture)				

	Ninohe (Iwate)	Yokote (Akita)	Saku (Nagano)	Ishikawa (Okinawa)	Katsushika (Tokyo)
Deepfried/Oily (“Kotteri”)	16.1	21.9	17.2	27.8	19.8
Hot	15.4	13.4	13.7	15.2	19.6
Salty	11.0	13.6	11.7	9.4	8.1
Sour	44.2	28.6	32.5	27.6	30.9
Sweet (eg. confectionery)	36.5	37.8	32.8	39.2	39.7
Hot (temperature)	41.3	33.4	30.5	39.3	28.1

**Table 3c.** Food Preferences (percent), cohort II, males.

	Public Health Center District (Prefecture)						

	Kasama (Ibaragi)	Kashiwazaki (Niigata)	Tosayamada (Kochi)	Arikawa (Nagasaki)	Miyako (Okinawa)	Suita1 (Osaka)	Suita2 (Osaka)
Oily (“Abura-koi”)	25.4	26.3	16.2	18.3	29.4	25.1	18.5
Salty	23.7	23.3	18.3	21.6	26.9	26.9	20.2
Sour	22.1	22.8	27.0	19.2	23.3	26.1	23.8
Sweet (dishes)	14.7	12.2	14.1	15.5	12.6	11.3	13.4
Sweet (confectioneries)	24.7	25.5	24.2	32.1	31.1	21.1	24.8
Hot (temperature)	21.6	19.7	18.8	25.4	25.8	28.3	24.7

**Table 3d.** Food Preferences (percent), cohort II, females.

	Public Health Center District (Prefecture)						

	Kasama (Ibaragi)	Kashiwazaki (Niigata)	Tosayamada (Kochi)	Arikawa (Nagasaki)	Miyako (Okinawa)	Suita1 (Osaka)	Suita2 (Osaka)
Oily (“Abura-koi”)	14.4	12.1	8.8	9.7	17.3	12.1	8.9
Salty	9.8	13.5	5.3	9.7	7.2	8.1	6.6
Sour	29.3	34.6	34.7	22.1	28.5	33.3	30.9
Sweet (dishes)	17.8	17.1	12.2	16.4	12.0	13.0	13.4
Sweet (confectioneries)	36.0	38.5	35.1	37.7	34.4	39.6	36.2
Hot (temperature)	27.9	28.0	28.1	35.5	33.1	35.8	30.1

### Cooking methods (Tables 4a, b, c, d)

﻿﻿For meats, “grilled” and then “stir fried” were two common cooking methods in most areas except Okinawa. When the preferences of two methods were compared between men and women, “grilled” were more preferred by men and “stir fried” by women. In Okinawa, “boiled” was the most commonly used cooking method. For sea foods, “grilled” was the most commonly used cooking method in most areas, but it was “boiled” and “raw” in Nagasaki, and “stir fried” in Okinawa. “Raw” was more commonly preferred by men than women. For vegetables, “raw”, “boiled”, and “stir fired” were three commonly used cooking methods in all areas except Okinawa. No consistent order was observed for these cooking methods in Cohort I. In Cohort II, in which “green vegetables” and “carrots” were separately asked in contrast to Cohort I which asked cooking method for all “vegetable”, “boiled” was the most commonly used cooking method in all areas except Miyako, Okinawa. “Raw” was more preferred by women than men. In Okinawa, “stir fried” was a far more commonly used cooking method than the other areas for vegetables.

**Table 4a.** Most commonly used cooking method (percent) for the following foods, cohort I, males.

	Public Health Center District (Prefecture)				

	Ninohe (Iwate)	Yokote (Akita)	Saku (Nagano)	Ishikawa (Okinawa)	Katsushika (Tokyo)
Meats					
raw	0.7	0.4	0.4	0.5	0.7
boiled	25.4	23.7	32.3	52.0	10.0
grilled	46.0	41.7	37.4	18.8	50.5
deep fried	7.4	11.7	12.0	6.0	7.6
stir fried	19.5	20.1	16.8	21.8	29.7
others	1.1	2.4	1.1	1.0	1.5
Seafoods					
raw	7.8	10.9	5.5	19.0	25.0
boiled	12.7	15.6	11.1	27.4	10.7
grilled	78.1	70.7	80.6	15.6	62.8
deep fried	0.6	0.7	1.5	34.4	0.7
stir fried	0.1	0.2	0.4	2.0	0.1
others	0.7	1.9	0.8	1.6	0.8
Vegetables					
raw	22.2	32.1	38.4	13.8	36.5
boiled	46.6	24.7	37.0	12.8	25.7
grilled	0.9	0.4	0.1	0.4	0.2
deep fried	0.8	2.2	0.3	0.3	0.3
stir fried	26.8	33.7	21.2	71.7	35.3
others	2.6	6.9	3.1	0.9	2.0

**Table 4b.** Most commonly used cooking method (percent) for the following foods, cohort I, females.

	Public Health Center District (Prefecture)				

	Ninohe (Iwate)	Yokote (Akita)	Saku (Nagano)	Ishikawa (Okinawa)	Katsushika (Tokyo)
Meats					
raw	0	0.1	0	0.2	0.2
boiled	24.2	21.5	26.1	57.5	9.2
grilled	35.2	31.4	29.4	13.9	45.1
deep fried	12.9	16.1	18.7	6.0	9.4
stir fried	26.0	27.8	23.5	21.3	34.6
others	1.7	3.1	2.1	1.1	1.4
Seafoods					
raw	1.9	3.1	1.4	11.2	10.8
boiled	13.1	17.8	13.2	27.3	16.4
grilled	83.5	76.0	82.8	17.1	71.2
deep fried	0.5	0.7	1.3	40.8	0.6
stir fried	0.2	0.2	0.3	2.1	0.3
others	0.7	2.2	1.0	1.6	0.7
Vegetables					
raw	17.8	25.1	33.8	8.7	21.0
boiled	53.2	32.0	39.9	10.6	47.3
grilled	0.9	0.2	0.2	0.3	0.1
deep fried	0.7	1.0	0.2	0.2	0.1
stir fried	25.5	35.8	23.4	79.0	29.9
others	2.0	5.9	2.5	1.1	1.6

**Table 4c.** Most commonly used cooking method (percent) for the following foods, cohort II, males.

	Public Health Center District (Prefecture)						

	Kasama (Ibaragi)	Kashiwazaki (Niigata)	Tosayamada (Kochi)	Arikawa (Nagasaki)	Miyako (Okinawa)	Suita1 (Osaka)	Suita2 (Osaka)
Meats							
raw	0.5	0.4	0.6	0.8	0.4	0.4	0.3
boiled	26.2	33.9	30.4	17.4	48.0	11.8	21.2
grilled	41.3	27.8	36.6	37.6	10.5	60.6	48.4
deep fried	6.4	6.1	3.7	13.0	4.4	4.0	4.1
stir fried	23.4	29.6	25.7	27.4	34.4	21	22.1
multiple responses	2.0	2.1	3.1	3.8	2.3	2.2	3.9

Seafoods							
raw	15.1	6.3	27.8	36.2	21.8	23	19.0
boiled	17.2	24.6	15.5	35.7	52.2	19.7	24.3
grilled	63.6	64.0	49.3	16.3	6.4	52.6	49.7
deep fried	0.9	1.2	1.6	2.2	10.6	1.1	1.0
stir fried	0.4	0.3	0.2	0.9	3.7	0.3	0.3
multiple responses	3.0	3.7	5.6	8.7	5.3	3.3	5.8

Green leafy vegetable							
raw	17.1	13.8	18.0	23.9	11.8	24.8	22.4
boiled	48.8	54.9	56.6	31.7	30.3	53.1	58.6
grilled	0.3	0.2	0.1	0.4	0.4	0.3	0
deep fried	0.5	0.3	0.3	1.0	0.9	0.2	0.2
stir fried	30.5	26.0	20.5	37.3	53.2	18.6	14.2
multiple responses	2.7	4.9	4.5	5.7	3.4	2.9	4.6

Carrot							
raw	4.4	4.6	3.8	4.4	1.3	6.0	6.7
boiled	73.9	70.5	74.3	57.8	35.2	66.2	69.5
grilled	0.3	0.2	0.2	0.3	0.5	0.9	0.4
deep fried	0.9	0.4	0.7	1.1	1.1	0.4	0.4
stir fried	18.6	20.8	18.4	32.6	59.6	24.2	19.6
multiple responses	2.0	3.6	2.6	3.8	2.4	2.4	3.4

**Table 4d.** Most commonly used cooking method (percent) for the following foods, cohort II, females.

	Public Health Center District (Prefecture)						

	Kasama (Ibaragi)	Kashiwazaki (Niigata)	Tosayamada (Kochi)	Arikawa (Nagasaki)	Miyako (Okinawa)	Suita1 (Osaka)	Suita2 (Osaka)
Meats							
raw	0.1	0.1	0.2	0.2	0.1	0.1	0
boiled	23.1	22.2	27.5	18.5	48.1	13.9	24.8
grilled	37.8	23.9	22.9	20.8	6.8	43.6	36.8
deep fried	7.5	7.3	5	15.7	5.3	6.8	4.6
stir fried	28.3	42.9	40.8	39.8	36.2	32	29.8
nultiple responses	3	3.6	3.7	5.3	3.5	3.6	4

Seafoods							
raw	5.5	0.8	14.1	14	11.4	7.4	7.7
boiled	23.3	29	20.1	52	57.8	23.5	27.4
grilled	65.9	63.7	58.2	21.2	6.9	62.9	58.1
deep fried	1	0.4	1.8	3.4	14.4	1.5	0.8
stir fried	0.4	0.3	0.2	0.7	3.1	0.4	0.5
nultiple responses	3.9	5.8	5.7	8.6	6.3	4.3	5.4

Green leafy vegetable							
raw	10.8	8.6	9.9	13.2	8.3	10.9	8.6
boiled	56.7	48.3	59.7	32.8	29.1	69.6	72.2
grilled	0.2	0.1	0.1	0.3	0.5	0	0
deep fried	0.2	0.4	0.1	0.2	0.3	0	0
stir fried	28.5	37.3	24.4	46.8	57.4	15.4	14.1
nultiple responses	3.6	5.4	5.8	6.7	4.4	4.2	5

Carrot							
raw	4.3	3.3	4	2.8	0.8	5.3	5.7
boiled	73.4	60.6	66.9	52.5	30.4	66.2	66.5
grilled	0.2	0.1	0.2	0.2	0.3	0.1	0.1
deep fried	0.7	0.3	1	0.5	0.5	0.1	0.3
stir fried	17.9	30.2	23.6	37.8	64.1	24.7	21.5
nultiple responses	3.6	5.5	4.4	6.1	3.9	3.6	5.8

### Participation on Dietary advice (Tables 5a, b, c, d)

﻿﻿No remarkable regional difference was observed in the percentages of acceptance to dietary advices. For all questions, the percentage of followers was higher for women than men.

**Table 5a.** Participants on Dietary Advice (percent), cohort I, males.

	Public Health Center District (Prefecture)				

	Ninohe (Iwate)	Yokote (Akita)	Saku (Nagano)	Ishikawa (Okinawa)	Katsushika (Tokyo)
To decrease consumption of burnt part of fish or meat	61.3	66	57.4	75.9	57
To decrease consumption of salty foods	70.6	73.8	67.5	79.2	56.4
To decrease consumption of foods rich in cholesterol	57.3	63	56.2	67	44.2
To increase consumption of green and yellow vegetables	82.3	87.1	84.3	90.6	74.9
To decrease consumption of animal fats	62.5	66.7	58.3	72	54.7

**Table 5b.** Participants on Dietary Advice (percent), cohort I, females.

	Public Health Center District (Prefecture)				

	Ninohe (Iwate)	Yokote (Akita)	Saku (Nagano)	Ishikawa (Okinawa)	Katsushika (Tokyo)
To decrease consumption of burnt part of fish or meat	74.3	82	79.1	85.7	80.6
To decrease consumption of salty foods	84.2	85.8	82.4	88.5	78.3
To decrease consumption of foods rich in cholesterol	67.7	71.8	69.5	77.1	59.5
To increase consumption of green and yellow vegetables	91.9	95.2	93.4	95.8	91.5
To decrease consumption of animal fats	73.6	80	74.3	83.9	73.7

**Table 5c.** Participants on Dietary Advice (percent), cohort II, males.

	Public Health Center District (Prefecture)						

	Kasama (Ibaragi)	Kashiwazaki (Niigata)	Tosayamada (Kochi)	Arikawa (Nagasaki)	Miyako (Okinawa)	Suita1 (Osaka)	Suita2 (Osaka)
To avoid consumption of burnt part of fish or meat	56	46	59.9	68.4	75.3	46.5	55
To decrease consumption of salty foods	68.2	70.3	71.5	67.3	78.1	54.3	70.1
To decrease consumption of foods rich in cholesterol	57.1	49.1	58.7	58.9	61.9	45.4	63.4
To increase consumption of green and yellow vegetables	84.3	89.1	84.1	82.9	90.9	76.4	81.2
To decrease consumption of animal fats	67.4	62	72.9	65.9	66	53.7	72.6

**Table 5d.** Participants on Dietary Advice (percent), cohort II, females.

	Public Health Center District (Prefecture)						

	Kasama (Ibaragi)	Kashiwazaki (Niigata)	Tosayamada (Kochi)	Arikawa (Nagasaki)	Miyako (Okinawa)	Suita1 (Osaka)	Suita2 (Osaka)
To avoid consumption of burnt part of fish or meat	76.4	71.6	77.1	81.6	84.7	67.2	69.5
To decrease consumption of salty foods	85.1	84.5	89.3	82.5	87.7	76.6	85.9
To decrease consumption of foods rich in cholesterol	77.1	68.9	76.9	75	75.9	67.6	82.1
To increase consumption of green and yellow vegetables	93.7	95.6	93	91.3	94.6	88.6	90.7
To decrease consumption of animal fats	80.9	77.8	84	78.6	77.7	68.7	85.1

## DISCUSSIONS

Geographic differences have been shown in several items in frequencies of food consumption and cooking method, while it was not remarkable in food preference and acceptance rate of dietary advice. The differences were commonly observed between urban and rural (Tokyo and Osaka vs. others) or Okinawa and non-Okinawa. The so-called westernized foods such as bread, beef and coffee were more consumed in urban areas such as Tokyo, Osaka and Okinawa. The frequencies of salted food intake such as pickled vegetables and salted seafoods were remarkably low in Okinawa. Cooking methods for meats, seafoods and vegetables were unique in Okinawa. These geographic differences were almost the same as those observed in the previous ecological study^8^^)^, in which high fat/low carbohydrate diets in Tokyo and Okinawa, and low sodium intake in Okinawa were reported^9^^)^.

The percent contribution of inter-population variance to the total variation (inter- plus intra-population (within- and between-individual)) was relatively notable in fat (9%) and sodium (10%) by three day dietary record survey conducted in five Cohort I areas^10^^)^. This feature may facilitate ecological studies in nutrient and disease at the population level, however several unmeasurable population characteristics may confound at individual-level analysis in a cohort study.

In the baseline survey of JPHC Study, we only assessed food frequency using four or five category and portion size was only provided in certain number of foods in Cohort II. This dietary assessment method has several limitations not only with respect to nutrient level analysis but also well-balanced ranking for 100,000 cohort members. We therefore developed a semi-quantitative food frequency questionnaire (SQFFQ) based on the dietary record data in Cohort I areas^11^^)^ and applied it at the 5 – and 10 – year follow-up survey in the entire JPHC Study area. The validity of this SQFFQ has been testing based upon the 7-day dietary records in four seasons from 30 couples each among participants in 11 JPHC study areas and furthermore these data are analyzed in conjunction with biomarkers in blood and collected urine from the same individuals.
